# Facile one-step synthesis of TiO_2_/Ag/SnO_2_ ternary heterostructures with enhanced visible light photocatalytic activity

**DOI:** 10.1038/s41598-018-28832-w

**Published:** 2018-07-12

**Authors:** Zewu Zhang, Yuhang Ma, Xiaohai Bu, Qiong Wu, Zusheng Hang, Zhao Dong, Xiaohan Wu

**Affiliations:** 1 School of Materials Science and Engineering, Nanjing Institute of Technology, Nanjing, 21167 P. R. China; 2 Jiangsu Key Laboratory of Advanced Structural Materials and Application Technology, Nanjing, 21167 P. R. China

## Abstract

Novel TiO_2_/Ag/SnO_2_ composites were successfully prepared by a facile one-step reduction approach using stannous chloride as both SnO_2_ precursor and reducing agent. The Ag nanoparticles with sizes of 2.04–3.94 nm were located on TiO_2_ matrix and immobilized by the surrounded SnO_2_. The resulted TiO_2_/Ag/SnO_2_ nanocomposites were used as photocatalyst for photodegradation of methylene blue under visible light. The experimental results demonstrated that the visible light photocatalytic activity of the TiO_2_/Ag/SnO_2_ was significantly enhanced in comparison with the individual TiO_2_ or the binary composite (TiO_2_/Ag or TiO_2_/SnO_2_) and the degradation rate was up to about 9.5 times that of commercial TiO_2_. The photocatalytic activity of the TiO_2_/Ag/SnO_2_ composites could be well controlled by simply tuning the dosages of Ag precursor and the optimized activity of the composites was obtained when the dosage of Ag precursor was 2%. Moreover, the TiO_2_/Ag/SnO_2_ photocatalyst exhibited high stability for degradation of methylene blue even after four successive cycles.

## Introduction

Currently, photocatalysis applications for addressing environmental issues such as environment pollution and energy crises have attracted more and more attention and gradually become a research hotspot^1–4^. In most case, non-toxic, chemically stable, controllability of redox power through materials engineering, and the capable of retrieval and extended use without substantial loss of activity are often recognized as the rubric of semiconductor photocatalysis^5–7^. However, conventional homogeneous photocatalysis have inherent drawbacks such as the easy recombination of photo-induced electron-hole (e^−^/h^+^) pairs and the absorption of light only at ultraviolet region (λ < 400 nm)^8^. Developing heterogeneous photocatalysis has been an effective strategy to enlarge the range of wavelengths of light absorption and promote the separation of the charge-carrier^9,10^. Therefore, the heterogeneous photocatalysis often shows more appealing than its homogeneous counterpart.

Among the multi-heterogeneous systems that have been developed, TiO_2_-based photocatalysts have triggered considerable interest due to their unique photocatalytic activity and good chemical stability^11^. Heterostructures of TiO_2_ and other oxides, such as ZnO^12^, SnO_2_^13–15^, and Fe_2_O_3_^16^ to form the semiconductor coupling is believed to overcome the facile recombination of e^−^/h^+^ pairs. In the suitably assembled semiconductors, the efficient charge transfer can be occurred, ultimately leading to the spatially separation of the charge-carrier. In particular, the SnO_2_/TiO_2_ system with high photocatalytic activity has attracted extensive interest. On one hand, SnO_2_ possessed a high electron mobility (~100–200 cm^2^V^−1^s^−1^)^17^, which gives rise to a faster transport of photoinduced electrons. On the other hand, the conduction band (CB) edge of SnO_2_ is more positive than that of TiO_2_^18^, which may lead to the transformation of photoexcited electrons from the CB of TiO_2_ to that of SnO_2_, and the opposite transformation direction for holes in the heterojunction between TiO_2_ and SnO_2_^19^. By this way, the recombination of charge carriers can be greatly suppressed, thereby resulting in an enhanced photocatalytic performance.

Additionally, in regard to the weak visible light response, decorating TiO_2_ with noble metal nanoparticles(NPs) to construct the noble metal/TiO_2_ composite was suggested to be an effective method to extend the photoresponse of TiO_2_ to visible light region due to the localized surface plasmon resonance (SPR) for metallic nanoparticles^20^. In these heterogeneous systems, the noble metal NPs can be excited by visible light in the ways that the oscillating electric field of the light interacts with the conduction electrons^21^. As a result, a strong oscillation of these electrons appears when the incident photon frequency is comparable to the collective oscillation of the conduction electrons^22^. Still now, there are several methods have been reported for incorporation of noble metal NPs into TiO_2_, such as adsorption of preformed noble metal colloids^23^ and photo-deposition^24^. But unfortunately, all of these strategies are suffered from the weak interaction between TiO_2_ and noble metal NPs, and also the problem of inhomogeneity distribution of noble metal NPs^25^. As is well known, the strong linkage of noble metal NPs to TiO_2_ may enhance the electron transfer between noble metal NPs and TiO_2_ and can also prevent the metal NPs to leach from TiO_2_ surface^26^. Moreover, the surface homogeneity distribution of small noble metal NPs might increase the density of metal/TiO_2_ interface. All of these enhance the photocatalytic activity of the composites comprised with the pure TiO_2_. In these regards, it is necessary to develop a facile strategy to prepare the composite photocatalyst that coupled with noble metal NPs, SnO_2_ and TiO_2_.

Recently, our group^27^ reported a convenient Sn^2+^ reduction method to prepare a ternary heterostructure TiO_2_/SnO_x_-Au photocatalyst and the photocatalyst exhibited an enhanced visible photocatalytic performance as compared with TiO_2_/SnO_2_ and TiO_2_/Au binary composites. In this catalytic system, the Au nanoparticles were connected with SnO_x_ surface directly, which may weaken the interaction with noble metal NPs and TiO_2_ matrix inevitably. In this regard, it may be more meaningful to construct a ternary heterostructure in which noble metal NPs were coupled with both TiO_2_ matrix and tin oxides accelerant.

**Figure 1:**
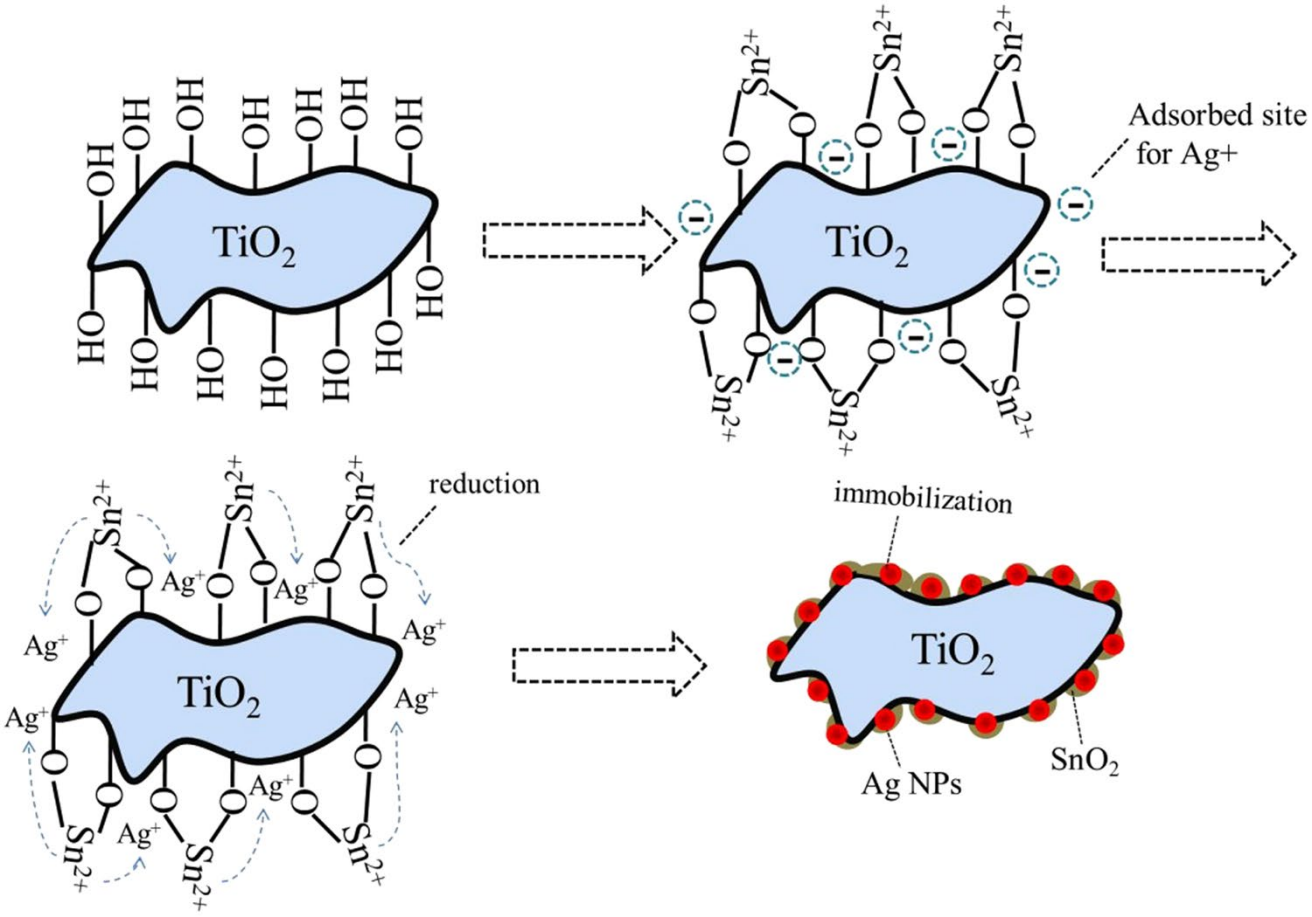
Methodology used for the preparation of the TiO_2_/Ag/SnO_2_ photocatalysts.

In the present work, we have constructed a ternary heterostructure TiO_2_/Ag/SnO_2_ photocatalyst with Ag nanoparticles by SnO_2_, by which we want to maximize the potential of Ag NPs for extending the visible-light absorption and the SnO_2_ species for inhibiting the recombination rate of photo-generated h^+^/e^−^ pairs. The preparation route was shown in Fig. 1. The Ag NPs in the ternary composites exhibited a quite uniform distribution with the particles size could be facilely tuned from 2.04 nm to 3.94 nm. The ternary TiO_2_/Ag/SnO_2_ composites offered an enhanced catalytic activity for degradation of methylene blue under the visible light irradiation as compared with the single TiO_2_ and the binary hybrid materials (TiO_2_/Ag and TiO_2_/SnO_2_).

## Experiment

### Synthesis of TiO_2_/Ag/SnO_2_ composites

In a typical experiment, 1.0 g of P25 TiO_2_ was dispersed in deionized water (100 mL) by ultrasonic processing. Then a mixture aqueous solution containing SnCl_2_ (0.5 g) and hydrochloric acid (3.0 mL) were added into the above solution, which was allowed for stirring at room temperature for 12 h. The precipitate was collected by centrifugation, followed by washing with water and redispersed into 70 mL water. Subsequently, 0.5 mL of AgNO_3_ solution (50 mM) was added. After reaction for 30 min, 2 mL of 0.15 M sodium formate solution was added. The mixture was allowed to stir for another 4 h, and then the product was collected by centrifugation, washing with water and drying in a vacuum oven at 75 °C. The obtained samples were labeled as TiO_2_/Ag/SnO_2_(x wt%), where the x denoted the nominal content of Ag NPs in the ternary composites.

### Characterization

Transmission electron microscopy (TEM) experiments were conducted on a JEM-1230 microscope operated at 100 kV. The samples for the TEM measurements were suspended in ethanol and supported onto a Cu grid. The powder X-ray diffraction (XRD) patterns were recorded on a Bruker D8 Advance Diffractometer (Germany) with Cu Kα radiation (λ = 1.5406 Å). X-ray photoelectron spectra (XPS) measurements were carried out in a Thermo ESCALAB 250 instruments (USA) using non-monochromatic Al Kα1486.6 radiation. The nitrogen adsorption and desorption isotherms were measured at −196 °C on an ASAP 2020 (Micromertics USA).The specific surface area was determined from the linear part of the BET equation (P/P_0_ = 0.05–0.25). The pore size distribution was derived from the desorption branch of the N_2_ isotherm using the Barrett-Joyner-Halenda (BJH) method. UV-vis spectra were recorded on a Shimadzu UV 3600 spectrometer.

### Evaluation of photocatalytic performance

40 mg TiO_2_/Ag/SnO_2_ composites were added into 100 mL of 3.12 × 10^−5^ mg/L methylene blue (MB) solution. A 500 W xenon lamp, the main wavelength of which lies in the 365–720 nm range, was used as the visible light source. Before irradiation, the solution was stirred for 30 min in dark in order to achieve absorption-desorption equilibrium. Then, the aforementioned mixture solution was irradiated in a photochemical chamber under continuous stirring with reflux water to keep the temperature constant. At certain time intervals, 2 mL solution was drawn out and centrifuged to obtain clear liquid. The quantitative determination of MB was performed by measuring the intensity of its absorption peak with a UV-vis spectrophotometer.

## Results and Discussion

The TiO_2_/Ag/SnO_2_ ternary composites were prepared followed by the strategy containing a facile one step reduction approach by using stannous chloride as both SnO_2_ precursor and reducing agent. The commercial Degussa P25 TiO_2_ composed with 80% anatase and 20% rutile was used as the support due to its good application prospect^28^. In the first step, the TiO_2_ particles were activated with Sn^2+^ by the inorganic grafting between Sn^2+^ and surface hydroxyl groups on TiO_2_ particles^29^. Secondly, AgNO_3_ solution was added into the TiO_2_/Sn^2+^ species. It is well known that the isoelectric point of Degussa P25 TiO_2_ is about 6.2^30^. Therefore, the surface of TiO_2_ is possessed of negative charge in the neutral environment. Except the part of the TiO_2_ surface being neutralized by Sn^2+^, the residual position with negative charge can be served as the adsorption site for self-assembly of Ag^+^. Since the standard reduction potential of the Sn^4+^/Sn^2+^ (0.151 V *vs*. redox pair the standard hydrogen electrode, SHE) is lower than that of Ag^+^/Ag(0.80 V *vs*. SHE), the deposited Ag^+^ can be easily reduced to Ag NPs at ambient temperature by the surrounding Sn^2+^ species, with the Sn^2+^ species being oxidized to SnO_2_. As a result, Ag NPs were incorporated onto TiO_2_ surface and isolated by the SnO_2_ species.

**Figure 2:**
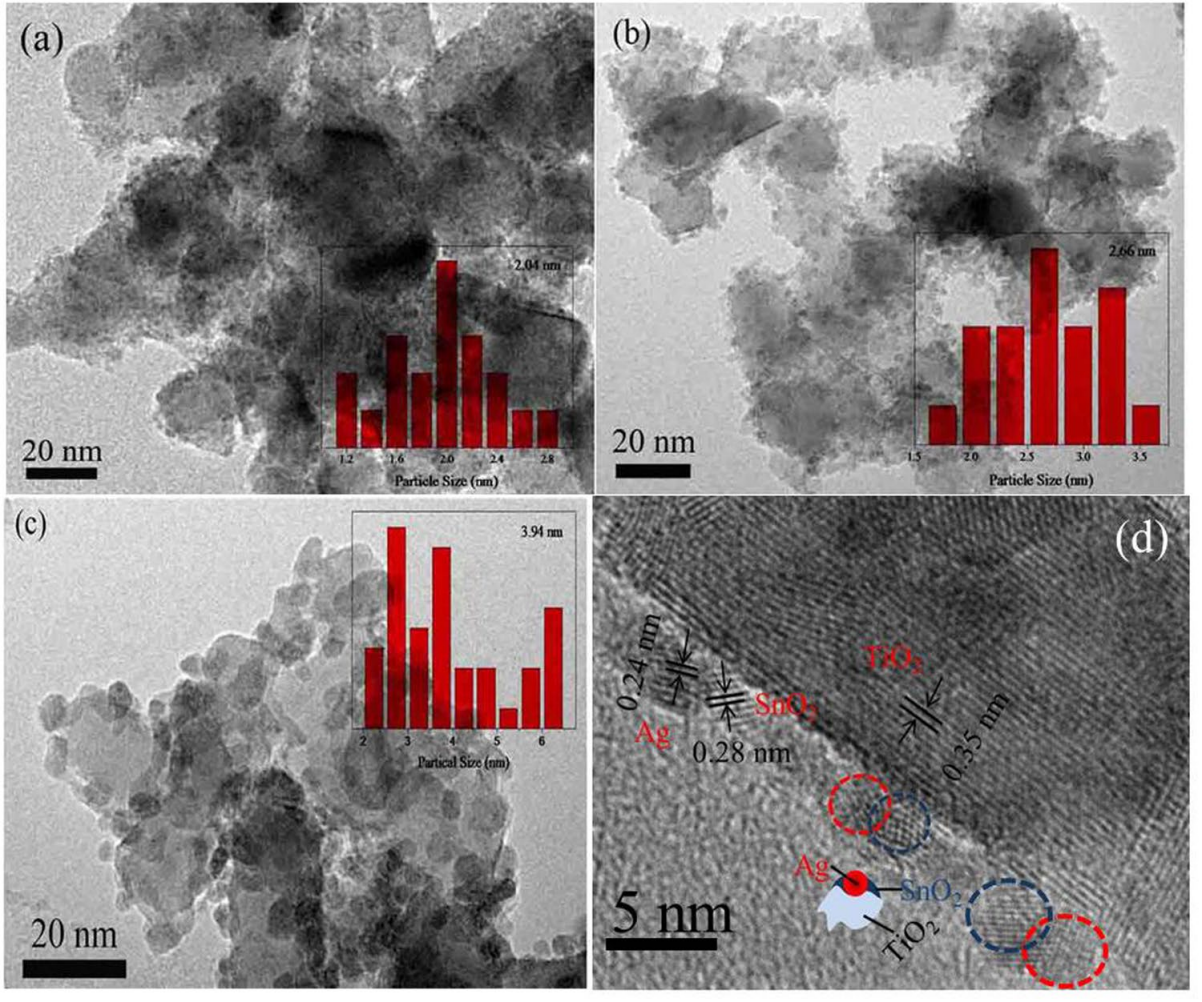
TEM images of as-prepared TiO_2_/Ag/SnO_2_ photocatlaysts with different Ag contents: (**a**) 1 wt%, (**b**) 2 wt% and (**c**) 5 wt%. (**d**) HRTEM images of TiO_2_/Ag/SnO_2_(2 wt%) photocatlaysts.

Figure 2(a) showed the TEM image of TiO_2_/Ag/SnO_2_ photocatalyst with the nominal Ag content was 1 wt%. It was obviously that the Ag NPs with the average particle size of about 2.04 nm were well-distributed on the TiO_2_ surface (with the size range of Ag NPs is 1.1 nm~2.9 nm). We attributed it to the evenly distribution of Sn^2+^ species on TiO_2_ that gives rise to the uniform adsorption sites for Ag^+^. As a result, the Ag NPs were homogeneously distributed in the ternary composites. Interestingly, the particles size of the Ag NPs on the support could be tuned by facilely changing the dosage of AgNO_3_ (Fig. 2(b,c)).When the nominal dosage of AgNO_3_ was 2 wt%, the average size of the formed Ag NPs increased to ~2.66 nm (with the size range of Ag NPs is 1.5 nm~3.6 nm). Further increase the nominal concentration of Ag to 5 wt%, the Ag NPs possessed a larger particles size (~3.94 nm, with the size range of Ag NPs is 2.0 nm~6.4 nm), suggesting that the particle size of the incorporated Ag NPs on TiO_2_ surface could be easily controlled. It should be mentioned that the SPR effect is strongly related to the content and particle size of noble metal NPs^31^, therefore it seems that the convenient adjustment of Ag NPs is crucial for optimizing the photocatalysis. But beyond that, it should be mentioned that Ag NPs in all the heterostructure composites exhibited uniform distribution even with the growth of Ag NPs. Figure 2(d) displayed the typical HRTEM images of the TiO_2_/Ag/SnO_2_(2 wt%) photocatlaysts. The image indicated that the ternary composites were composed of Ag NPs located on TiO_2_ matrix and immobilized by the surrounded SnO_2_.

**Figure 3:**
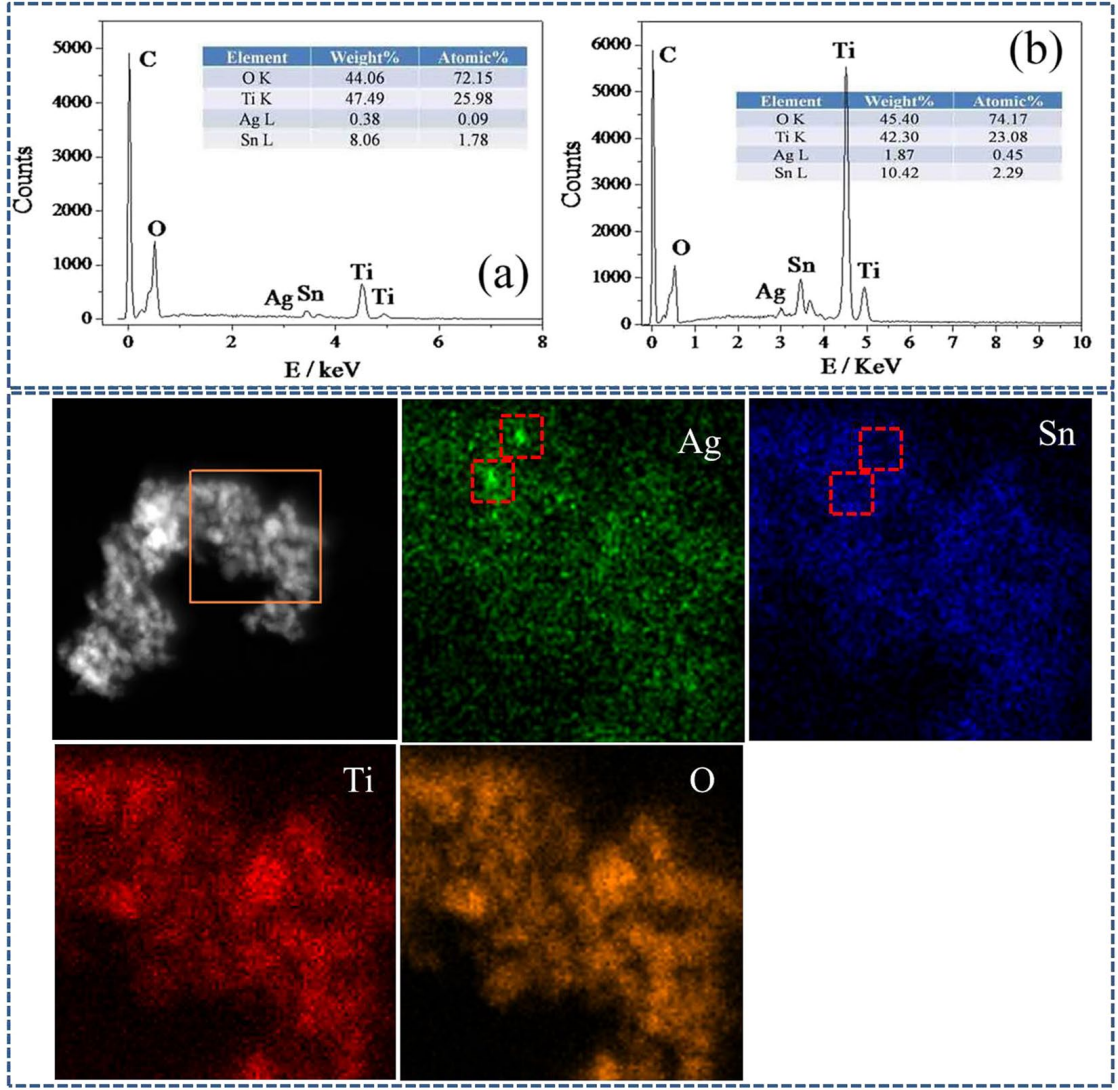
TOP: EDX analysis of (**a**) TiO_2_/Ag/SnO_2_(1 wt%) and (**b**) TiO_2_/Ag/SnO_2_(5 wt%). (**c**) A representative EDS-Mapping of TiO_2_/Ag/SnO_2_(5 wt%). Down: HAADF-STEM image of the as-obtained TiO_2_/Ag/SnO_2_(2 wt%).

Figure 3 displayed the energy dispersive X-ray (EDX) spectroscopy of TiO_2_/SnO_2_/Ag photocatlaysts. The result showed that the content of Ti, O and Sn in all samples is almost the same, suggesting that the incorporation of Ag NPs in the photocatalysts have little influence on the content of TiO_2_ and Sn species. Additionally, the Ag content in TiO_2_/SnO_2_/Ag samples increased with the increased dosage of AgNO_3_, demonstrating that the concentration of Ag NPs can be easily tuned in our experiment. Figure 3 also displayed the HAADF-STEM of the TiO_2_/Ag/SnO_2_. Though the images appear blurry, it might be concluded that Ag nanoparticles were tightly covered by Sn species from the red frames in the mapping images of Ag(Ag-L) and Sn(Sn-L), which is bright in Ag images but dark in Sn images.

**Figure 4:**
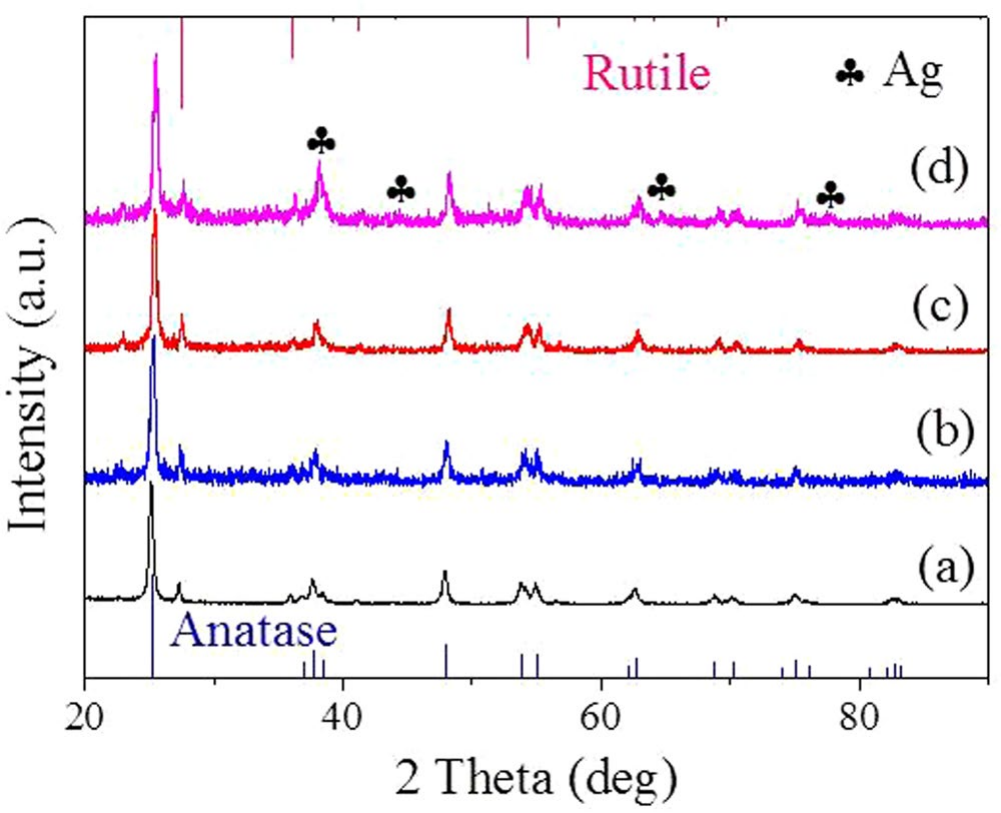
XRD patterns for (a)TiO_2_/SnO_2_, (b) TiO_2_/Ag/SnO_2_(1 wt%), (c) TiO_2_/Ag/SnO_2_(2 wt%) and (d) TiO_2_/Ag/SnO_2_(5 wt%).

XRD patterns of the TiO_2_/Ag/SnO_2_ samples were shown in Fig. 4. All the samples exhibited the mixed crystalline phase containing anatase and rutile, which is on account of the P25 TiO_2_ that used as the support in our experiment containing anatase and rutile phases^32^. The result also suggested that the loading of SnO_2_ or Ag had minor influence on the crystalline phase of original TiO_2_. No obvious Ag and Sn species peaks could be seen in the composites that incorporated 1 wt% and 2 wt% Ag, which might be attributed to the relatively low crystallinity of Sn species and the quite small Ag NPs, respectively^33^. As increasing the size of the supported Ag NPs to 3.94 nm(TiO_2_/Ag/SnO_2_(5 wt%)), the diffraction peaks that could be assigned to the Ag NPs with face-centered cubic structures (fcc) emerged.

**Figure 5:**
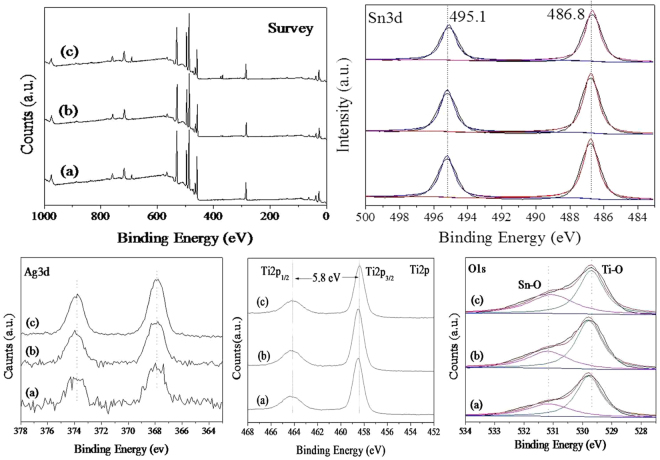
XPS patterns of (a) TiO_2_/Ag/SnO_2_(1 wt%), (b) TiO_2_/Ag/SnO_2_(2 wt%) and (c) TiO_2_/Ag/SnO_2_(5 wt%).

The chemical and bonding environments of the ternary composites were ascertained by XPS measurements. As shown in Fig. 5, the fully scanned spectra revealed that the presence of Ti, O, Sn and Ag in all the ternary TiO_2_/Ag/SnO_2_ composites. The high-resolution spectrum of the Ag 3d region in the TiO_2_/Ag/SnO_2_(5 wt%) displayed two peaks corresponding to metallic Ag at binding energies of 367.8 eV and 373.8 eV in Ag 3d_3/2_ and Ag 3d_5/2_ levels, with the splitting of the 3d doublet is 6.0 eV, revealing the complete reduction of Ag^+^ in the experiment^34^. It was obvious that the peaks of Ag 3d shifted to the lower position as compared to these of bulk Ag (368.3 eV for Ag 3d_3/2_ and 374.3 eV for Ag 3d_5/2_)^35^, indicating the increase in electrons density of Ag species and also revealing the strong interaction between Ag and the semiconductors^36^. Moreover, the binding energies of Sn 3d5/2 and Sn 3d3/2 are 486.8 and 495.1 eV, respectively, indicating that tin, in all samples, is in the +4 oxidation state^37,38^. In the experiment, Sn^2+^ was completely oxidized by the excess Ag^+^, and the formed Sn^4+^ species could not be easily reduced by sodium formate^39,40^. As a consequence, it is reasonably to calculate that the Sn species in TiO_2_/Ag/SnO_2_ ternary heterostructures are dominated by the SnO_2_. The Ti 2p spectrum in TiO_2_/Ag/SnO_2_(1 wt%) can be ascribed to Ti 2p3/2 and Ti 2p1/2 that centered at binding energies of 464.3 and 458.6 eV correspondingly. The splitting of the binding energies was ~5.7 eV, which indicated that the typical Ti^4+^ in the composite sample^41,42^. The Ti 2p spectrum do not vary with the increase the content of Ag NPs, suggesting that the incorporation of higher quantity of Ag have few influence on the chemical environments of TiO_2_.We attributed it to the lager amount of TiO_2_ species in the composites that could offer abundant electrons without the alteration of the chemical state of TiO_2_ particles.

**Figure 6:**
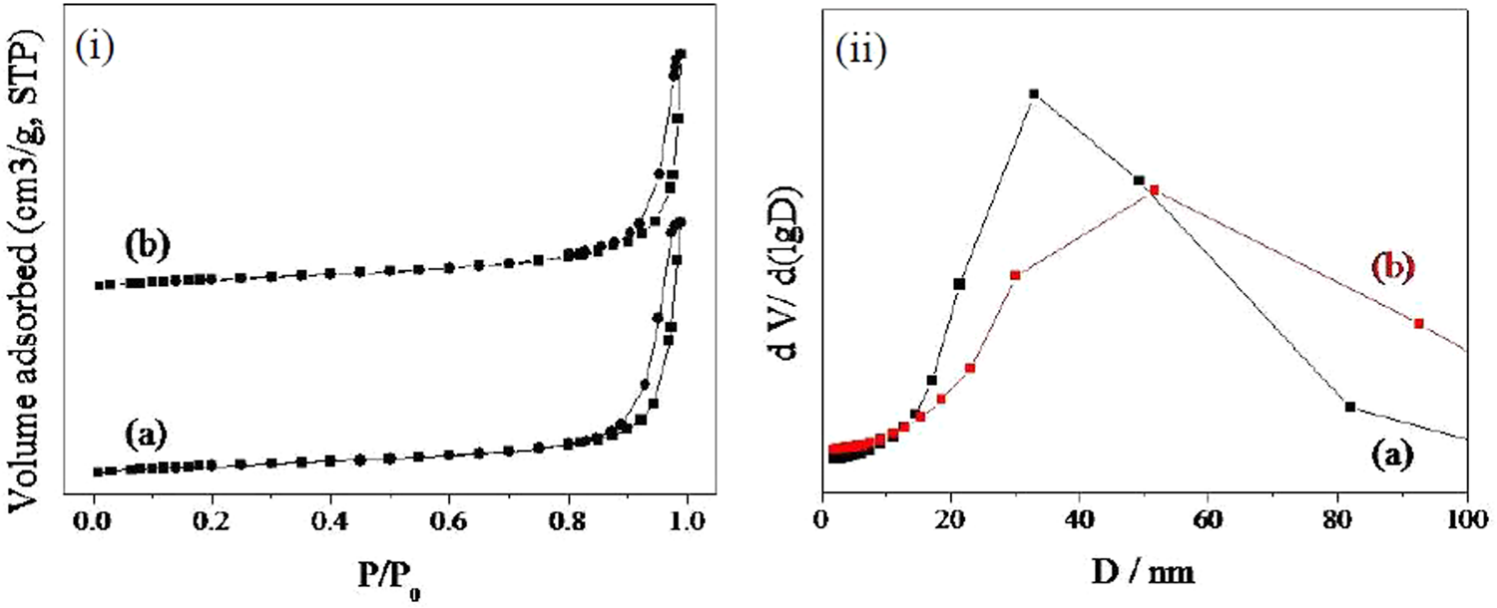
N_2_ adsorption-desorption isotherm and corresponding pore size distribution curve (inset) of (a) TiO_2_/Ag/SnO_2_(1 wt%), and (b) TiO_2_/Ag/SnO_2_(5 wt%).

The nitrogen adsorption-desorption isotherm and corresponding size distribution of the as-prepared TiO_2_/Ag/SnO_2_ samples were shown in Fig. 6. It was obviously that all the TiO_2_/Ag/SnO_2_ samples exhibited the typical porous characteristics, as evidenced by the significant type IIb curves in the N_2_ absorption-desorption isotherm of TiO_2_/Ag/SnO_2_ sample^43^. This pore should be ascribed to the space among the stacking of the grain in the TiO_2_ matrix or the SnO_2_ shells. Additionally, the feature of the isotherm was not be changed as the alteration of Ag content, indicating that Ag NPs were mainly deposited on the surface of TiO_2_ instead of filling into the pores of TiO_2_ matrix. The same result could also be verified from the pore size distribution of the sample in Fig. 6(ii), which showed the similar pore size distribution of the two samples, with the average pore size being ~40 nm.

**Figure 7:**
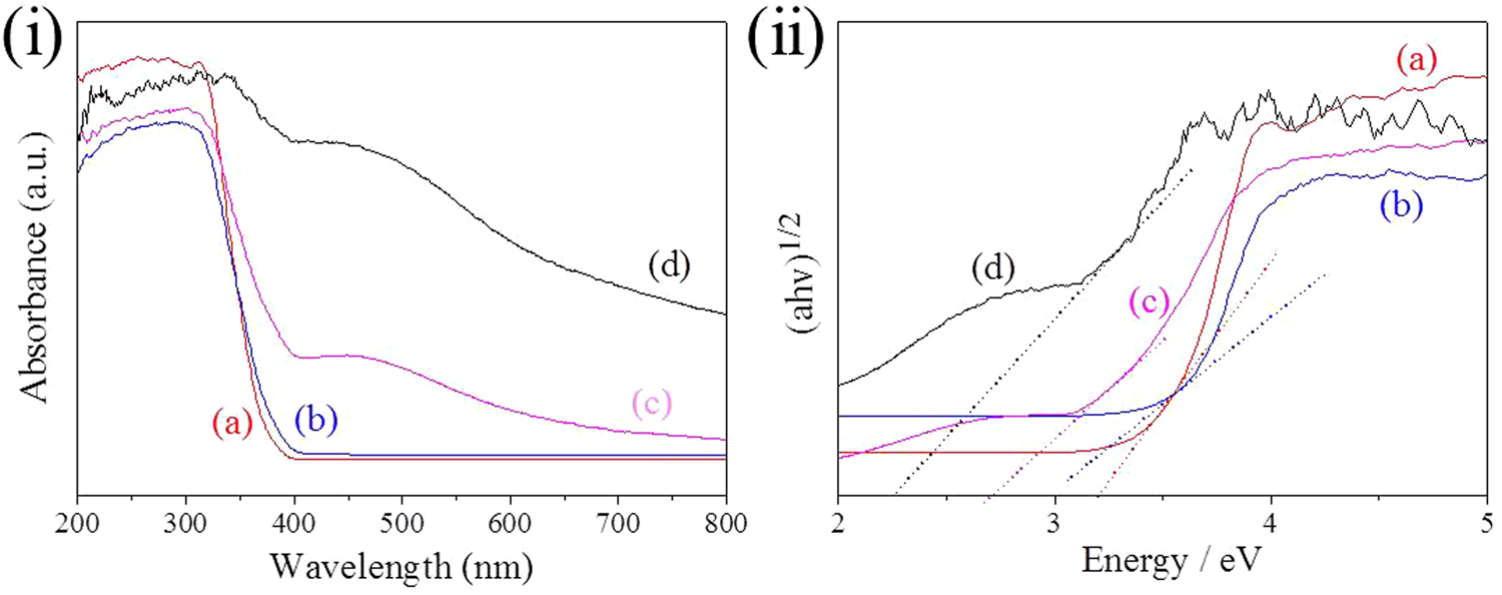
(**i**) UV-vis diffuse reflectance spectra and (**ii**) the corresponding Tauc plot of (a) original TiO_2_, (b) TiO_2_/SnO_2_, (c) TiO_2_/Ag/SnO_2_(1 wt%) and (d) TiO_2_/Ag/SnO_2_(2 wt%).

The optical properties of the as prepared samples were characterized by UV-vis DRS, and the results were shown in Fig. 7. Compared with the unmodified TiO_2_ nanoparticles, the absorption measurements of the TiO_2_/Ag/SnO_2_ sample exhibited enhanced photoabsorption in the range of 400–650 nm, which can be attributed to the light-harvesting enhancements by the surface plasmon resonance of Ag NPs^27^. Moreover, the absorption edge of the TiO_2_/Ag/SnO_2_ composite extended an unambiguous red-shift compared to the TiO_2_ and TiO_2_/SnO_2_, which reflected that the electronic structure and optical properties of the TiO_2_ and TiO_2_/SnO_2_ have been modified by the incorporated Ag species. The band-gap energy of the samples can also be determined from the plot of (ahν)^1/2^ versus hν (Fig. 7(ii))^44^. The optical band gap energy of the TiO_2_/Ag/SnO_2_(1 wt%) composite was measured to be 2.7 eV, which was relatively lower than that of TiO_2_(3.2 eV) and TiO_2_/SnO_2_(3.0 eV). These result also confirmed that the incorporation of Ag and SnO_2_ caused a red shift of the UV-Vis absorption spectrum and narrowed the optical band gap energy of TiO_2_. Moreover, the TiO_2_/Ag/SnO_2_(2 wt%) composite exhibited a higher absorption and an even narrower band gap energy (2.3 eV) than the TiO_2_/Ag/SnO_2_(1 wt%) composite, possibly suggesting that the enhanced electron transfer existed among the components of TiO_2_/Ag/SnO_2_(2 wt%). Benefiting from such broad light absorption width and high absorption intensity in the visible region, the ternary composite photocatalyst was expected to improve the solar-energy utilization efficiency and perhaps possessed an enhanced visible-light-driven photocatalytic performance.

### Visble-light photocatalytic activity

**Figure 8:**
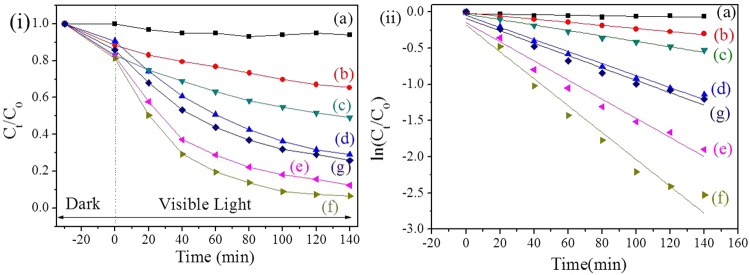
Photocatalytic activities (**i**) and kinetics (**ii**) of the different catalysts. (a) No catalyst, (b) TiO_2_, (c) TiO_2_/SnO_2_, (d) TiO_2_/Ag, (e) TiO_2_/Ag/SnO_2_(1 wt%), (f) TiO_2_/Ag/SnO_2_(2 wt%), and (g) TiO_2_/Ag/SnO_2_(5 wt%) for degradation of MB under visible light irradiation.

The photocatalytic performances of the as-prepared TiO_2_/Ag/SnO_2_ composites were evaluated by monitoring its characteristic absorption band at 650 nm to measure the degradation rate of MB under visible light irradiation. Before irradiation, the reaction mixtures were stirred in dark for 30 min to ensure that the adsorption-desorption equilibrium of MB was established. As shown in Fig. 8(i), the ternary TiO_2_/Ag/SnO_2_ heterostructure exhibited a slightly increase adsorption efficiencies for MB (~10%) as compared with the commercial TiO_2_ and binary heterostructure(TiO_2_/Ag, and TiO_2_/Sn^2+^), demonstrating that the incorporation of Ag and Sn species can increase the adsorption of MB.

The photodegradation of MB does not occur without the presence of photocatalysts, as evidenced by little change in the absorption peak after visible light irradiated for 2 h. After addition of a trace amount of photocatalyst into the solution, the adsorption peak significant decreased, suggesting the degradation of the organic pollutant proceeded. Figure 8(i) showed plots of C_t_/C_0_ in the degradation of MB, where C_0_ is initial concentration of MB and C_t_ is the concentrations of the MB at time t, respectively. It is undisputed that the degradation rate of MB followed the order of TiO_2_/Ag/SnO_2_ > TiO_2_/Ag > TiO_2_/SnO_2_ > TiO_2_ after the same irradiation time. The result indicated that the degradation efficiency of MB could be improved in the presence of Ag or SnO_2_ modified TiO_2_ photocatalytic systems as compared to pure TiO_2_, particularly remarkably enhanced with the Ag/SnO_2_ co-decorated TiO_2_ photocatalyst. Furthermore, the photocatalytic degradation of the organic pollutant could be regarded as a pseudo-first-order kinetics reaction to evaluate the degradation rate. The linear relationships between ln(C_t_/C_0_) and reaction time using the samples were shown in Fig. 8(ii), and the plots of all the samples were well matched the first-order reaction kinetics. The calculated rate constant k for TiO_2_, TiO_2_/SnO_2_,TiO_2_/Ag and TiO_2_/Ag/SnO_2_(2 wt%) samples were 0.002 min^−1^, 0.004 min^−1^, 0.008 min^−1^ and 0.019 min^−1^, respectively. It is apparent that TiO_2_/Ag/SnO_2_(2 wt%) exhibited the best degradation efficiency among the above four samples, giving a 9.5 times higher rate constant of MB degradation than the commercialize TiO_2_.

As discussed before, the excellent photocatalytic performance for the TiO_2_/Ag/SnO_2_(2 wt%) sample, on one hand, should be largely attributed to the SPR effect of Ag NPs induced broadband optical absorption enhancement. The porous characteristic of the ternary composites may also promote the connection between the embedded Ag NPs and the external environment, leading to a strong SPR effect of Ag NPs. On the other hand, it could be ascribable to the SnO_2_ species may serve as an electron tank to accept the photogenerated electrons and facilitate charge carriers separation^45^. Interesting, as compared the rate constant with that of TiO_2_/SnO_x_/Au reported previously(0.014 min^−1^)^27^, it could be found that the k values of the samples(0.019 min^−1^ for TiO_2_/Ag/SnO_2_(2 wt%)) even exhibited an increased photocatalytic activity. In the previous report, it was suggested that the electron trapping capability of Au was excellent than that of Ag upon the higher electron affinity of Au NPs, which inevitably give rise to a higher photocatalytic activity for the Au modified photocatalysts^46^. In this work, the abnormal higher activity of TiO_2_/Ag/SnO_2_ can be attributed to the intimate connection among the Ag, SnO_2_ and TiO_2_. In our experiment, the Ag NPs were initially absorbed on TiO_2_ surface, and reduced by the surrounding Sn^2+^ species, rustling in Ag NPs directly located on the TiO_2_, and anchored by the SnO_2_ species. This structure may allow the maximum improvement level of interaction between each component of the photocatalyst, resulting in an enhanced photocatalytic performance. While for the TiO_2_/SnO_x_/Au, the Au NPs were located on the SnO_x_ surface, which may weaken the interaction between noble metal NPs and TiO_2_ matrix. Indeed, comparing the rate constant with that of Au-based and Ag-based photocatalysts reported previously listed in Table 1, it can be found that the activity of TiO_2_/Ag/SnO_2_(2 wt%) in our experiment is much higher than that of most reports for the photodegradation of MB.

**Table 1:** Comparison of rate constant for the photodegration of MB using catalysts containing Ag nanoparticles.

photocatalyst	photocatalyst concn (mg)	initial MB concn (10^−5^ M)	degradation rate (10^−2^ min^−1^)	ref
Au/Ag/TiO_2_	0.03	3	1.06	^50^
Ag/ZnO	150	0.5	0.55	^51^
Ag_2_Mo_2_O_7_/Ag	50	3.13	0.23	^52^
TiO_2_/SnO_2_/Au	40	3.12	1.4	^27^
TiO_2_/Ag/SnO_2_	40	3.12	1.9	this work

Besides, one can see that the TiO_2_/Ag/SnO_2_ heterostructure catalysts with Ag content 2% revealed the highest photocatalytic activity. When the Ag content is relatively low(<2%), the photodegration of MB enhanced gradually with increase of the Ag content, which may be attributed to the increase of the electron transfer interface both of Ag-TiO_2_ and Ag-SnO_2_. However, when the Ag content exceeded 2%, an opposite phenomenon was observed by further increase the content of Ag. The lower photocatalytic performance should be ascribed to the re-combined electrons and holes upon the excess content of Ag NPs.

**Figure 9:**
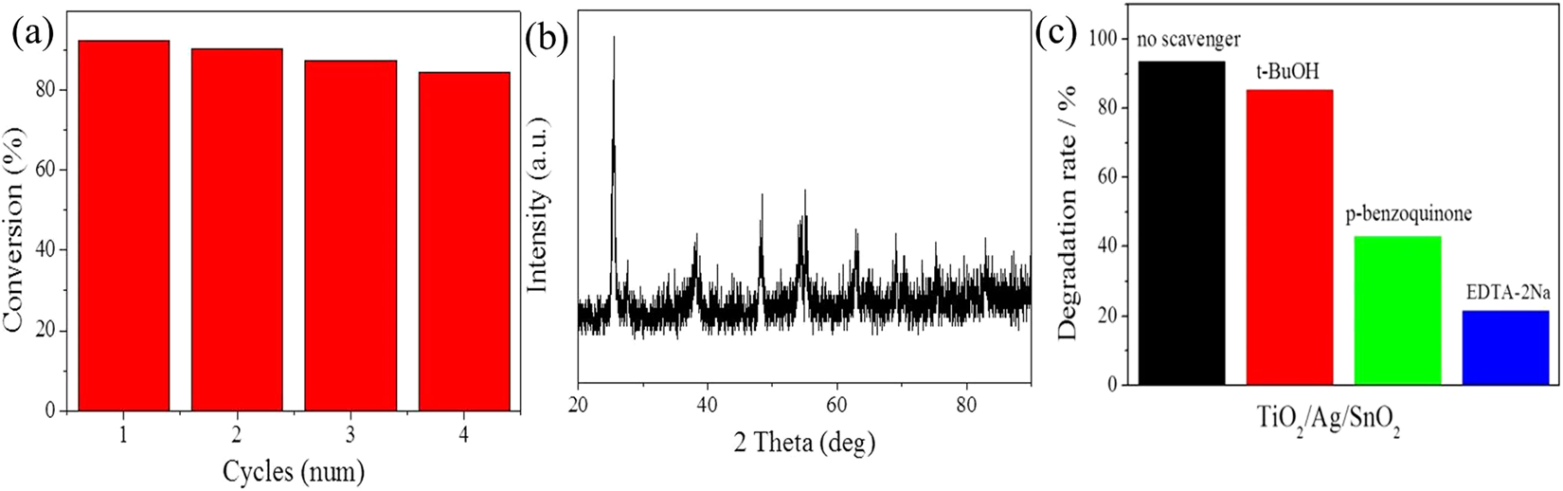
(**a**) Conversion of 4-NP in 4 successive cycles of reduction using TiO_2_/Ag/SnO_2_(2 wt%) as photocatalyst, (**b**) XRD pattern of the TiO_2_/Ag/SnO_2_(2 wt%) catalyst after reaction, (**c**) Effects of a series of scavengers on the degradation efficiency of MB by TiO_2_/Ag/SnO_2_(2 wt%) photocatalyst.

Because the practical application of photocatalyst requires its renewable, we carried out four cycling tests to degrade MB using the as-prepared TiO_2_/Ag/SnO_2_(2 wt%) photocatalyst to investigate its stability, as shown in Fig. 9a. It is observed that the TiO_2_/Ag/SnO_2_(2 wt%) sample only exhibited a slight decline for photocatalytic decomposition of MB shows after four cycles(9.1%). Almost no changes can be found form the XRD pattern of catalyst after reaction (Fig. 9b), demonstrating the excellent stability of the catalyst.

**Figure 10:**
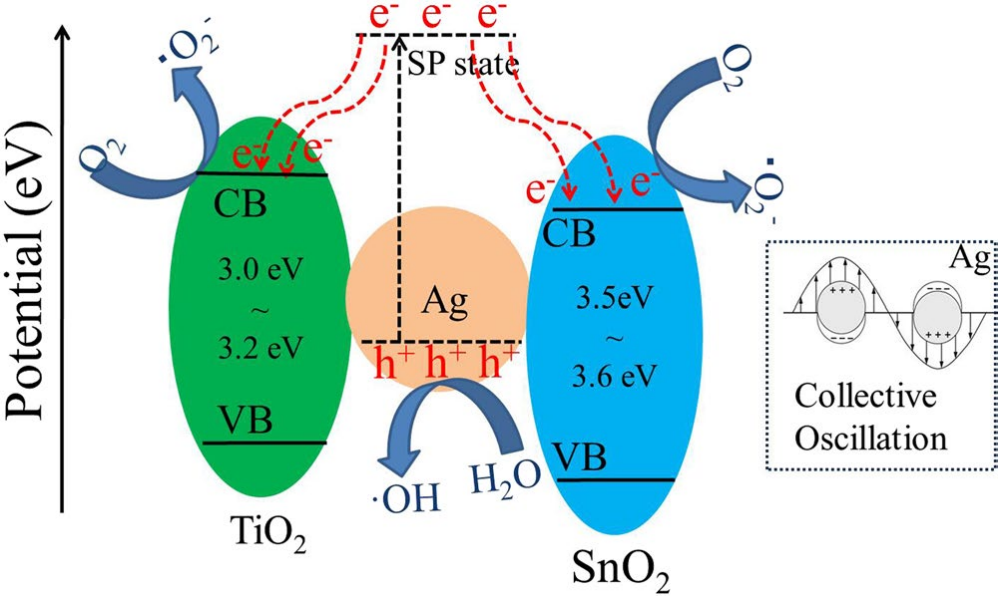
Proposed photocatalytic mechanism for degradation of MB by TiO_2_/Ag/SnO_2_ nanocomposites under visible light irradiation.

Based on the above experimental results and discussion, a possible mechanism for degradation of MB by visible-light-driven TiO_2_/Ag/SnO_2_ photocatalyst was proposed and presented in Fig. 10. Under visible light irradiation, only the Ag SPR can be excited, resulting in the emerging of electrons in the CB while the holes (h^+^) remain in the VB of Ag NPs^47^. These photo-induced electrons can get sufficient energy to surmount the Schottky barrier on Ag/TiO_2_ and Ag/SnO_2_ and move into the CB of semiconductor (TiO_2_ or SnO_2_) through their tightly-coupled interfaces, where the electrons can be consequently trapped by the adsorbed molecular oxygen and produce superoxide radicals(▪O_2_^−^)^48^. At the same time, the h^+^ on the VB of Ag in turn accept electrons from water or the dye molecules adsorbed on the surface of catalysts, resulting hydroxyl radicals(▪OH) generating in the surface of Ag NPs^49^. These reactive oxygen species and h^+^ are potent oxidizing agents for the degradation of methylene blue molecules. In order to distinguish the roles of the active species, we have taken the trapping experiment with scavenger investigation. Three reagents, t-BuOH, p-benzoquinone and EDTA, were used as the scavengers of •OH, •O_2_^−^ and *h*^+^, respectively. Figure 9c showed the degradation rates of MB by TiO_2_/Ag/SnO_2_(2 wt%) in the conditions of adding scavengers. When t-BuOH (5 mM) was added into reaction solution, the photocatalytic efficiency was slightly reduced to 85.3%. However, the photocatalytic efficiency exhibited a significant decrease with the addition of p-benzoquinone (5 mM) or EDTA (5 mM), and the degradation rates of MB were reduced to 42.9% and 21.6%, respectively. Obviously, the results suggested that •O_2_^−^ and h^+^ are the main active species in the current photocatalytic system. The unique structure of TiO_2_/Ag/SnO_2_ with Ag NPs tightly immobilized on TiO_2_ by SnO_2_ species may promote the interactions between Ag and the semiconductor (TiO_2_ and SnO_2_), which can accelerate the separation of photo-induced holes and electrons. As a result, the holes and electrons can be entirely involved in the photocatalytic reactions, and a strong photocatalytic activity is expected.

## Conclusions

In summary, the ternary TiO_2_/Ag/SnO_2_ heterostructure composites were successfully prepared by a facile one-step reduction approach using SnCl_2_ as both SnO_2_ precursor and reducing agent. The Ag^+^ was first adsorbed on TiO_2_ surface and then reduced by the surrounding Sn^2+^ species, resulting in the formation of the TiO_2_/Ag/SnO_2_ composite. The obtained TiO_2_/Ag/SnO_2_ heterostructure exhibited enhanced photocatalytic activity toward MB degradation under visible light irradiation as compared to individual TiO_2_ or the binary composite (TiO_2_/Ag or TiO_2_/SnO_2_). The significantly improved photocatalytic activity should be attributed to the SPR effect of Ag NPs and also the fast charge separation by the formation of tightly connected interface of TiO_2_/Ag and SnO_2_/Ag. Moreover, the TiO_2_/Ag/SnO_2_ composite exhibited good visible-light photocatalytic stability and reusability.
